# Assistive devices to support daily time management of persons with dementia: the Indian experience

**DOI:** 10.1002/alz.095235

**Published:** 2025-01-09

**Authors:** Sebestina Anita Dsouza, Kshama Bangera, Vinita Acharya

**Affiliations:** ^1^ Manipal Academy of Higher Education, Manipal, Karnataka India

## Abstract

**Background:**

Persons with dementia (PwD) have difficulties in managing time for daily activities due to cognitive impairments. This affects their participation and adds to caregiver burden. Research in developed countries has reported beneficial effects of assistive devices to support daily time management (DTM) of PwD. There is limited research on the use of these devices for dementia care in low‐ middle‐ income countries. To address this gap, a pre‐post interventional study was undertaken in India where individuals aged 60 years and above with mild to moderate dementia having self or caregiver reported DTM difficulties were provided with assistive devices including low‐ and high‐tech devices. The present study was a part of the main study to understand the experiences of PwD and their caregivers of using the devices.

**Methods:**

The study entailed a qualitative approach and involved 12 caregivers and three PwD purposively selected from the main study. The participants were interviewed individually using an interview guide at the time of reassessment after using the devices for three months.

**Results:**

The qualitative data suggested that the devices facilitated PwD’s orientation to time, daily activities, and routines, and reduced annoyance or anger associated with reminders from caregivers. Participants appreciated features of some devices such as simple and easy operation, portability, and customization. They reported concerns such as the need for continuous power supply and cueing from caregivers and device specific issues such as complex operationality, and cumbersome controls. They suggested the inclusion of Indian languages, pictures, and time format to enhance cultural relevance. The data also suggested that devices should be provided considering the personal characteristics of the PwD and caregivers and cultural factors such as family support and attitudes.

**Conclusions:**

The findings inform occupational therapists of factors influencing the provision, acceptance, and use of assistive devices by PwD to support DTM. The findings would also help in the design and development of contextually relevant, affordable. and accessible assistive devices in the Indian context.

